# Women’s Empowerment and Health: A Narrative Review

**DOI:** 10.3390/ijerph21121614

**Published:** 2024-12-02

**Authors:** Marina Couva, Michael A. Talias, Miranda Christou, Elpidoforos S. Soteriades

**Affiliations:** 1 Healthcare Management Program, School of Economics and Management, Open University of Cyprus, Nicosia 2220, Cyprus; michael.talias@ouc.ac.cy (M.A.T.);; 2 Department of Education, University of Cyprus, Nicosia 2109, Cyprus; 3 Environmental and Occupational Medicine and Epidemiology (EOME), Department of Environmental Health, Harvard T.H. Chan School of Public Health, Boston, MA 02115, USA

**Keywords:** empowerment, women, patriarchy, gender bias in health

## Abstract

Empowerment, the process by which a person is enabled to increase control over decisions concerning their life, is a multidimensional construct that has been extensively discussed by various disciplines for more than four decades. Several empowerment models have been presented, based on different approaches. This paper proposes a four-step model, based on individual and contextual awareness and advancement. Disparate factors may positively or negatively affect empowerment, including gender, race, culture, education, financial autonomy, socioeconomic status, family, neighborhood, religion, social cohesion, civic society, and political context. Empowerment has been extensively discussed in parallel with health promotion, since it is expected to positively affect health, both on the level of the individual and community, as well as in the context of the patient–healthcare professional relationship. Considering the position of women in patriarchal societies, where women may experience feelings of powerlessness, their social position and more importantly health may be adversely affected. Gender biases that were developed due to the marginalized position of women in different societies, coupled with paternalistic approaches of healthcare professionals, may significantly contribute to higher comorbidity, albeit longer life expectancy for women. Empowerment can therefore be a powerful tool for achieving equity in health and improving women’s well-being.

## 1. Introduction

Historically, the need for empowerment has emerged from the fact that humans stratified to specific gender and race societal strata have been oppressed as a result of ruling powers exerted on them over the years, leading to discrimination and alienation, rendering them powerless. Facilitating the awareness of their situation and guiding them to take action toward self-determination were the first steps in the establishment of the empowerment theory [1,2]. The background of this oppression has been exposed and discussed extensively in Marxist, neo-Marxist, and post-colonial theories, covering the spectrum of exploitation of low social class and/or non-Western populations. None of the above-mentioned theories on oppression, which were the result of male scholars, have discussed the gender parameter that emerged only by feminist theorists that have been exclusively women until today. Therefore, focusing on women, the fact that for years they were regarded as objects in relation to men, who regarded themselves as the subject [3], has resulted in women being denied power and, consequently, the decision-making process concerning their own fates and lives. Over the years, this oppression became deeply embedded in social norms and people’s consciousness resulting in a permanent feeling of disempowerment [2,4].

Power is a force exercised by the subject producing intended effects on others [5]. This power is exerted through a superiority, either actual or perceived, that could have its origins in gender, race, ethnicity, wealth, etc. On an organized level, political decisions and policymakers may also be limiting the ability of individuals to make decisions for themselves [6]. Examining the circumstances under which people need support, by developing their ability to take control over their lives, may be a more efficient way to help them eventually become less reliant on their caregivers and/or institutions [7,8]. The oppressed can gain their freedom and enjoy their rights as human beings, arguing that this freedom cannot be given by oppressors but rather can be achieved by involving the oppressed in the process, through both education and action, leading them to conscientization, a status where they are fully aware of their oppressed state, and they are determined to act toward liberation, a sequence that is thought to instigate empowerment in the process [1,2].

Adopting a feminist perspective on the concept of empowerment, initially, Beauvoir declared “that one is not born but rather becomes a woman” implying that gender is a social construct, in a manifesto aiming to change the position of women [3]. A few decades later, the work of Michel Foucault discussed power relations in a gender-neutral way without even provoking the notion of the repressed claiming to liberate themselves from such relationships, even when there is violence involved [9]. Moving to the deconstructive approach of Butler, she supports that both sex and gender are fluid, arguing that the gender perspective may even influence sex and that the aim of Western medicine to normalize human bodies is a major cause of repression [10]. The role of contemporary medicine exercising a subtle form of power is also brought forward by Foucault, where again the female body experience is not discussed [9]. Taking into account that most of the research of modern medicine has been conducted by male doctors, who as subjects have tried to normalize their observation as per their experience [10], it does not come as a surprise that this medical knowledge available for several years in the Western world has not taken into account what women have been experiencing on a physical, mental, and emotional level. Even if this was a legitimate bias [11] that was realized in the last few decades, it resulted in women receiving significantly suboptimal treatment for several morbidities, to the extent that one can safely declare that being a woman is considered a risk factor [12].

The Marxist and post-colonial theories that brought forward oppression and the subsequent work of Freire, together with the feminist theories discussing women’s repression, laid the foundation for the discussion of women’s empowerment and health, which will be further addressed in this review.

Empowerment is considered a multidimensional construct that has been extensively discussed for more than 40 years in several contexts. The need was initially identified from the fact that institutions rendered people powerless [7], whereas supporting people to take action and improve their situation was considered better practice than prevention, when it came to treating patients with mental health issues [8]. This initial discussion on the possible use of empowerment in social psychology and public health followed the theory on the education of the oppressed in a way that would break this power cycle that formulated people as passive victims, a theory allowing them, through awareness, to claim their freedom by taking the responsibility to do so [1]. One may argue that people are in favor of making their own decisions; however, it is frequently seen that people who are oppressed, due to being part of certain social groups, may face the ‘fear of liberation’, a reluctance and a feeling of inability to decide for issues that are affecting their lives [1].

## 2. Definitions of Empowerment

Empowerment is grammatically a noun that is used to describe both a process and its outcome, a noun that derives from the word ‘power’ but does not necessarily mean the handing over of power. When attempting to define empowerment, one comes across a diversity of definitions listed in dictionaries and associated publications, thus verifying the complexity and broad spectrum of the dimensions and levels that contribute to this extensively discussed construct. Based on the etymological definition, it can be either considered the granting of power or authority to a person or the process where a person is enabled to make their own decisions, but still, there is no universally acceptable definition [13]. The fact that there is no clear, single definition of empowerment is what actually adds to its value, given that there is space for adjustment in the context of women’s empowerment [4]. In the same context though, the vagueness of the idea of empowerment has significantly affected the effectiveness of women’s empowerment programs with particular application in developing countries [14].

The lack of clarity in the definition of empowerment has led to further scientific discussions and debates in the way that empowerment is defined but also in the way that it can be measured, failing to result in a consistent and universally accepted way to capture its parameters [15,16,17,18].

Stressing the fact that empowerment is a ‘social construct’, Wallerstein [17] defines it as “a social-action process that promotes participation of people, organisations and communities towards the goals of increased individual and community control, political efficacy, improved quality of community life and social justice”. Kieffer [19], on the other hand, defines empowerment as “a developmental process from powerlessness to participatory competence”. This indicates a dynamic long-term development from socio-political illiteracy or “infancy” to socio-political “adulthood”. According to Gutierrez [2], there are three connotations of empowerment, one coming from the political approach, one from the psychological, and a third that is a combination of the other two. The provided definition stresses the political aspect, describing it as “the process of increasing personal, interpersonal, or political power so that individuals, families, and communities can take action to improve their situation”, a situation that mostly results from being marginalized due to gender or ethnicity [2,20].

Another approach to defining the way people exercise power is the so-called “power over”, which describes the situation in which a force is imposed upon people, as opposed to the “power from within”, referring to a condition where a person decides to take action on their own initiative, and the combination of the two, the “power with”, which actually defines the power flow in the context of empowerment [6].

The initial empowerment theory was proposed by Rappaport [8], where he claimed that “the aim should be to enhance the possibilities of people to control their own lives”, as a countermeasure of advocacy or prevention for solving social and community issues. Later on, he would define it as “a process, a mechanism by which people, organizations, and communities gain mastery over their affairs” [21] and further proceeded to correct himself that empowerment is a theory, a value, and a concept that can be applied at a personal, social, and organizational level and describes the process where a person gains control over their life [8,21].

Several scholars are still attempting to define empowerment, always as a multilevel construct comprising other distinct subconstructs, in an interactive relationship, resulting in a dynamic and circular process [15,18], and also proceed further to specifically discuss women’s empowerment as a separate construct, adjusted to the context resulting from the position of women [2,4,22].

Taking all the above, one may reach the conclusion that empowerment can be defined in several ways, but the one element that all above definitions have in common is the participation of the individual and their willingness to change, coupled with the environment that needs to enhance, support, and provide the necessary structural and organizational changes in order to provide related opportunities. Therefore, one can be either empowered on their own initiative or empowered with the support of others, and vice versa, a person may either empower themselves or others.

For the purpose of this paper, empowerment is defined as the process that enables people to gain control over their lives, leading them to a higher level of empowerment. One can never reach a state of absolute empowerment but will always follow a process, resulting in an outcome that will continue to support and feed the process. During this empowerment process, people may be negatively or positively affected by several factors, which will be further discussed in the next sections.

## 3. Measuring Empowerment

The challenges of measuring empowerment have also been repeatedly acknowledged in the scientific literature [14,16,17,22]. A possible explanation for this difficulty has been attributed to the different forms that empowerment can take, depending on the conditions relating to the individual and their environment [23]; therefore, there is no universal way to measure empowerment [18].

The adversity faced by many scholars during their attempts to measure empowerment may be possibly due to the way this multidimensional construct is approached. The proposed methodology for a more effective measurement suggests using the “formative” approach, where causality is directed from the individual constructs toward the multilevel construct, instead of the other way around. This bottom-up approach will result in an empowerment measurement that is based on the definition of sublevels and how these are defined and measured [16].

In separating the empowerment process from the actual outcome of the process, Zimmerman [18] proposed three domains for measuring empowerment. The intrapersonal domain relates to the perceived mastery of the individual, the way one understands their competencies, and the level of control they have over their lives. The interpersonal domain shifts from personal to “environmental mastery” and refers to the person’s understanding of their surroundings in a critical way and building the required abilities. Under this domain, skills related to leadership and decision-making can be found, as well as options for the deployment of available resources. The third and last domain is the behavioral domain, which stresses the importance of participation and focuses on people’s actions within their environment. Based on the above-described model, Cattaneo and Chapman’s [15] approach to empowerment follows that of an “iterative process” consisting of three steps: the first one is related to self-efficacy, where goals are defined; the second one is where action is taken; and during the third step, the obtained results are evaluated against the initially set targets.

Focusing on gender particularities, Jonhson [24] proposed a model for measuring women’s empowerment, the Personal Progress Scale Revised (PPS-R) that examines the following seven factors, related specifically to women’s empowerment: “perceptions of power and competence”, “self-nurturance and resource access”, “interpersonal assertiveness”, “awareness of cultural discrimination”, “expression of anger and confrontation”, “autonomy” and “personal strength and social activism”. Gram [22], focusing again on women’s empowerment, discussed four indicators that are used to measure women’s empowerment on a more global level, and these are decision-making, self-efficacy, relative autonomy index, and women’s collective empowerment. She further argues that these measures of empowerment can be “forward looking if they concern opportunities for future action or backward looking if they refer to a motivation behind a specific action”. Continuing with the gender perspective of black feminists, the importance of a woman’s experience should also be a part of the empowerment visualization, allowing each individual to define their self-actualization and self-determination [9].

**Figure 1:**
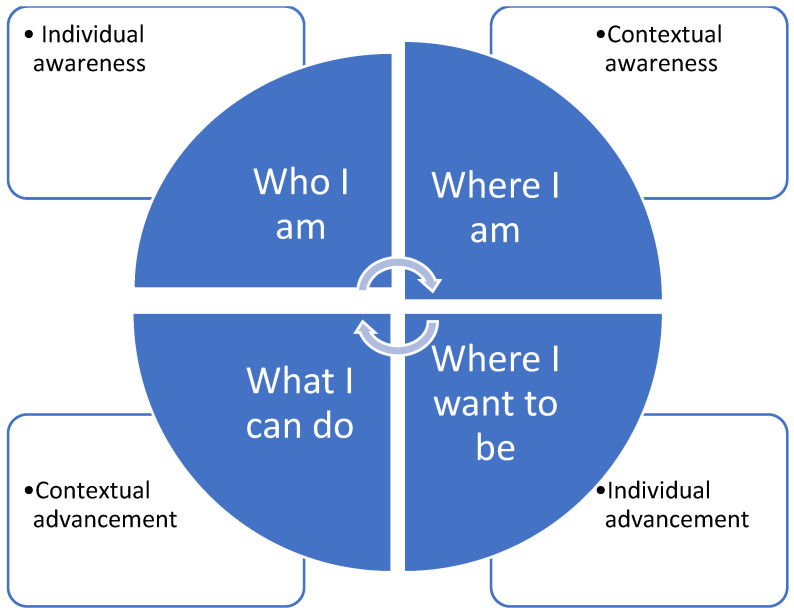
The proposed model of empowerment.

Based on the above, this paper proposes a model of empowerment that consists of four steps, two of them related solely to the individual and the other two related to the context in which the individual operates (Figure 1). The way several factors shape up the context and influence empowerment are described in subsequent sections of this paper.

Considering that empowerment is a continuous process, the first step would be the awareness of the individual regarding their personality traits, defined as “who I am”. The sublevel construct related to this step is self-esteem, indicating the extent to which a person values themselves, their perceived competence and self-efficacy, and their understanding of the level of control they currently have over their lives.

The second step involves “where I am”, which indicates the position of the person in their micro- and macro-environment, starting from their relationships with their close family and social networks and expanding to their society. Understanding power relations and the extent to which a person is in a position of powerlessness is part of this step, together with their ability to exhibit assertiveness and confront people in their direct environment who are in positions of authority. The effect of gender and race should also be taken into consideration.

Moving forward to the third step, the person should visualize “where I want to be” based on their own personal experience. At this phase, the level of satisfaction with their current situation should be identified, consideration of other options is encouraged, and willingness to shift to a situation where more decision-making freedom is attained be instigated. The change is visualized, and the person becomes aware that it is their own initiative to shift to a status where they have more control over their lives. By envisioning the change, they can feel motivated to proceed to the next step.

The last step is the “what I can do” phase of the process, where one should use the resources available to improve their life, reaching the point where empowerment is materialized. Participation and willingness to empower other people can be categorized under this domain, an action that evidently can induce feelings of empowerment in an individual. Challenges are accepted, and the fear of being in a situation of freedom is confronted.

The proposed model is currently theoretical and needs to be further validated through implementation.

## 4. Factors Influencing the Process of Empowerment

Empowerment comprises several constructs that spread on many levels and are influenced by many extrinsic factors, both on an individual and social level that may be considered either as barriers or facilitators. These factors can rarely be found as standalone, since they are frequently combined or intertwined, especially when discussing disempowering conditions, and they can be classified under broader categories of fixed or dynamic parameters.

### 4.1. Gender

Power has been deprived of women over the years, in patriarchal, male-dominating societies, where power was exerted onto women, depriving them of their right to self-determination [9,25,26,27]. Based on initial feminist theories, the independence of women is materialized first through their educational advancement, and economic and financial independence rather than through political independence. Taking into consideration that equality in legislation has been achieved in many countries throughout the 20th century, this did not necessarily lead to more women having decision-making positions [3]. Historically, gender is a factor of oppression, which may occur with other types of disempowering situations such as poverty or racial discrimination, resulting in the term ‘Intersectional Feminism’, defined by Kimberlé Crenshaw as “a prism for seeing the way in which various forms of inequality often operate together and exacerbate each other” [28]. Since individual empowerment is stronger and more efficient when it takes place in a favorable context, it can be significantly compromised in communities with strong patriarchal influence [26]. At the same time, the uneven distribution of power minimizes the access of women to the needed resources for better health, for themselves and their families [29]. This verifies that community and organizational empowerment are important contributing factors to achieving gender equality.

### 4.2. Race

There are racial and class barriers to the acquisition of power; therefore, what is sometimes achieved is the perception of empowerment rather than a real change to the actual status of a person or a group of people, posing a concern that the whole theory of empowerment may apply in an individualistic context and not consider the community aspects [23]. Women of color are considered to have a double disadvantage, including both their gender and race, and therefore empowerment is viewed as the solution for improving their individual and social status [2].

### 4.3. Culture

Considering the social, cultural, and political aspects of empowerment, saving the context is important, since the term has recently been downgraded to a popular word adopted by neo-liberal policies, resulting in a “transition of empowerment out of the realm of societal and systemic change and into the individual domain–from a noun signifying shifts in social power to a verb signalling individual power, achievement, and status” [30].

In parallel, participation was found to correlate more to the intrapersonal empowerment of African Americans, compared to the white population, meaning that African Americans would benefit more from community empowerment rather than the individual, compared to white Americans [31].

### 4.4. Economic and Financial Factors

Money equals power, and financial independence is a way of empowerment. Education can enhance a woman’s income, thus contributing indirectly to empowerment. Several examples of empowerment programs implemented in developing countries are available, where women are supported to start their own businesses in order to improve their quality of life and consequently improve their empowerment status, along with their health [32,33]. Nevertheless, feminist theorists question the context of empowerment interventions in developing countries by external agents, specifically the ones based only on economic advancement through microfinancing. They argue that this may happen in a neo-liberal environment that cannot be detached from the political and structural changes that need to be implemented in order to give women more decision-making power in practice [14].

### 4.5. Education

In the context of individual empowerment, education is an important facilitator [6]. Education, a strong social determinant of health and a vital parameter of socioeconomic status, contributes to several benefits that could be categorized as physical, psychological, and social. Further to contributing to mental development and unfolded opportunities, it can help people be more aware of health matters and adopt healthy behaviors. In addition, education provides a sense of control and empowerment [34]. The empowerment resulting from education is what has been defined as personal [6] or psychological [8], meaning that it results from the individual and can significantly enhance awareness, leading to a condition of conscientization [1]. Examining the effect of Egypt’s policy to increase access to higher education for women in the 1960s and 1970s, it was concluded that the policy led to their empowerment in both the workplace and their families [35]. Information is power, and new technologies can have a negative or positive effect on empowerment, depending on who controls the access and how they are implemented. For example, while the World Wide Web appeared to be an empowering tool, allowing information to reach all people, through the years, certain people have acquired more control over the Web and use it to exert power over others [36].

### 4.6. Socioeconomic Status

Socioeconomic status (SES) is a construct determined by income, education, and profession that combines some of the factors already discussed above, which can be significant empowering factors. Historically, low SES has been closely associated with feelings of powerlessness, which also results in poor health [17]. Although SES and empowerment are two distinct constructs, they are positively correlated, since the domains that comprise SES are all factors that can positively influence an individual’s empowerment process. It should be noted, however, that this positive correlation applies to the so-called psychological empowerment, which defines the empowerment of the individual. When the construct of community participation was measured, it was found to be negatively correlated with SES, indicating that people who were experiencing social injustice were more prone to take collective action to improve their situation [37].

Empowerment may refer to a perceived experience when the focus is on the psychological aspect, ignoring the social and structural changes that would indicate real and objective acquisition of power [13,38]. Correspondingly, this may simulate the extended discussion between the effects of relative and absolute socioeconomic status associated with the perceived and actual social ranking. The manifestation of empowerment is what has been controversially discussed in the literature, arguing whether working on empowering the individual without actual social and political changes would yield a sustainable result.

### 4.7. Family/Extended Family

Family, as an institution, is listed as one of the four structures that can support empowerment, supporting this claim on the crucial role of the family in the development of children into adulthood while acknowledging at the same time that family can be defined outside the man–woman norms [7]. On the other hand “sex/gender systems” are enforcing the gender roles in the family, thus suppressing women’s sexuality and forcing them to raise children that comply with these societal gender norms [27].

What has been observed through women’s empowerment programs in developing countries is that any increase in women’s income is used to improve the quality of life of the whole family [33]. Being responsible for their children’s well-being can help women be more empowered, while at the same time, the family itself may act as an agency of empowerment. In different cultural environments, the power relations within the family may vary, usually burdening women with financial dependence, the unpaid labor of housework, and childcare, as well as restrictions on their education and sexuality [32]. The possibility of women being severely abused within their own homes by their partners cannot be ignored; in those cases, empowerment is needed to help them take the necessary actions to liberate themselves from such situations, and many community organizations are providing support in this context [39].

### 4.8. Community and Neighborhood

The community where people live and socialize significantly impacts the level of control that people have, as well as their decision-making over their lives. A social context that breeds powerlessness will consequently result in poorer health, highlighting the social risk factors for disease and mortality. Therefore, by addressing these social parameters in an organized way, people may actually feel their level of awareness increase together with the ability to have more control over their lives, transforming their personal and social conditions [17].

The term “community empowerment” was then introduced in order to describe the many levels of the construct, thus denoting the importance of the environment surrounding the individual, summarizing, within the term, the many aspects that may characterize a community, such as common language, history, and culture [40,41]. Discussing the effect of the environment on the individual, Kieffer notes that “while not seen as unilaterally imposed on the individual by his/her environment, powerlessness is viewed as an experience embedded in and reinforced by the fabric of social institutions” [19], further stressing the importance that the community has on the construct. Community, in the form of neighborhood, has been identified as one of the four “mediating structures”, with the other three being family, religion, and voluntary organizations, that can promote public policies to actually bring empowerment down to the people [7].

### 4.9. Church and Religion

Empowerment in several cultures is also related to religion, both on an individual and community level, with the effect being controversial and significantly related to the religion or culture in which it is applied. The encountered paradox is that women practice religion with more diligence than men, whereas most religions are patriarchal, therefore somehow “encouraging” women’s repression. The phenomenon has been explored, leading to the conclusion that women use religion to empower themselves [42]. It is indeed a contradiction that, in general, religions do not support women’s emancipation, and therefore by considering that they should be subordinate to men, they deprive them of their freedom and equal rights, consequently undermining their empowerment. At the same time, it cannot be ignored that religious women can feel empowered by the love of God, which may help them to stand up for themselves and increase control over their lives [43].

An example of women who were significantly empowered by participating in religious communities, with positive effects on their health such as a reduction in cardiovascular risk among African American women, involves those who participated in a church-based intervention, compared to the group that was self-helped [44]. These findings support the fact that participation in a community, even a religious one, can be an empowering process [7].

### 4.10. Social Structure/Social Cohesion/Social Networks

This basic distinction between the person and the surrounding structure has been extensively discussed, and several similar theories have been developed. One of the fundamental ones supports that the empowerment of the individual, which is widely recognized as psychological empowerment, is not enough. In order for empowerment to be achieved, the environment plays also an important role; therefore, community and organizational empowerment were also acknowledged, stressing the importance of social and structural factors in achieving empowerment [8,17,21], otherwise described as ‘holistic’ [13], thus stressing how intertwined these two aspects are. The studies of Zimmerman and Rappaport [45] concluded that participation was positively correlated with empowerment, providing evidence for the importance of the environment in this construct.

Wallerstein [17] stresses the significance of the environment and the social context that may result in people lacking control and decision-making over their lives and consequently having poorer health, highlighting the social risk factors for disease and mortality. She further argues that “through challenging physical and social risk factors in a collective setting, people gain a belief they can control their world, a sense of their commonality, an ability to work together to acquire resources, and an actual transformation of socio-political conditions”.

### 4.11. Civic Society (NGO)

One of the eight Millennium Development Goals established in 2015 was the promotion of gender equality and women’s empowerment, with the main goal among the remaining ones being the eradication of extreme poverty, which is indirectly related to women’s powerlessness. The report denotes that following the adoption of the Beijing Platform for Action on women’s empowerment, there is a significant increase in the number of women in parliaments. The report concludes though that despite the fact that there has been an improvement, inequalities still exist [46]. In 2016, the Sustainable Development Goals (SDGs) were issued; among the 17 goals, the fifth one was to “achieve gender equality and empower all women and girls”. The SDGs are considered an improvement compared to the Millennium Development Goals since now a more holistic approach is being implemented [47,48].

### 4.12. Political/Legal

The significance of the political context of empowerment is stressed by the fact that the word power should be part of it, although there is the risk of leaving the meaning of empowerment empty of any political and social change; thus, losing its initial focus is a threat resulting from the recent overuse of the word [30]. In particular, in racial groups, the enforcement of ethnic realization resulted in higher political empowerment, based on studies on Latino students in the United States [20].

Empowerment has found many applications in several fields, ranging from education to sports, management, and health; the scope of this paper is focused on the latter.

## 5. Empowerment and Health

The empowerment concept found fertile ground in health, initially as a theory supporting better outcomes in mental health. The fact that therapists would not impose treatment on patients but rather encourage them to manage their psychological conditions in a dynamic and interactive way proved an effective method [8]. The characterization of ‘emerging technology’ for mental health [13] or the ‘Holy Grail’ of health promotion [49] captures and reflects the authors’ claim of the huge potential hidden in the empowerment theory.

Looking below the surface and seeing how the need to empower emerges leads us to the evaluation of dis-empowerment, better defined as powerlessness, which is a causal factor for poor health that goes beyond poor hygiene and lack of resources related to poverty [17]. Being in a situation in which one has low control, either perceived or actual, and being in a position with limited decision power result in chronic stress [50], which, in combination with the lack of resources and social support, leads to powerlessness [17]. Further to the studies of Karasek, the Whitehall study II extended the cohort to include issues related to women’s health, concluding that low income is not the only factor responsible for health inequalities. Organizational ranking and the performance of repetitive and low decision-making operations at one’s job can adversely affect their health [51]. Therefore, the reason that low socioeconomic status (SES) increases the risk of morbidity and mortality is not solely the lack of basic physical needs but interestingly the psychological impact associated with low societal resources, as well as the depression linked with individuals’ lack of decision power, affecting their everyday life [4,6,33,40,52]. However, compared to the studies supporting several poor health outcomes related to powerlessness, the evidence supporting an association of empowerment with better health outcomes is limited [17,49] and not so extensively explored. One example is a study from Minya, Egypt, where women who were able to make their own decisions had improved mental health, particularly reduced anxiety [53]. Nevertheless, empowerment has been scarcely studied with respect to improved health.

Empowerment can therefore be related to health under two major domains that are distinct but at the same time interrelated. One relates to the positive health effects resulting from empowerment, where the empowerment takes place outside the healthcare professional intervention, and they may be related to several intrinsic or extrinsic factors, as described in previous sections. The other domain tackles the relationship between the patient, who should take action toward improving their health, and the physician, who should empower patients to take control and ownership of their condition.

Initially, empowerment had its application in mental health [8], with therapeutic psychological interventions for abused women targeting their empowerment [15,24]. It then evolved into a useful tool for patient education in other medical disciplines. Programs that focus on patient compliance by minimizing patients’ decision-making may be less effective than programs focusing on empowering patients and increasing their self-confidence in the management of chronic diseases [54].

Using the concept of empowerment in the patient–doctor relationship could actually promote the understanding that the two parties are equal, and the physician is not treating the patient from a position of power. The paternalistic approach of many healthcare professionals focusing on enforcement may initially achieve good treatment results; however, in the long-term, patient empowerment proves to be a better option, specifically when treating type 2 diabetes mellitus, where a multidisciplinary approach is required, including weight management, exercise, and dietary habits [55,56]. When diabetic patients are enabled by their physicians to make their own decisions regarding the treatment of their condition, the results are more beneficial and sustainable for them, since they are based on their increased self-confidence in managing their disease [55].

During the implementation of health policies and protocols, patients’ empowerment may be compromised [7]. On the other hand, patient empowerment may be a convenient way for health systems to shift the responsibility to the patient, even in cases where the patients belong to marginalized populations that experience powerlessness due to their SES and/or gender and race [57]. The support provided by a closely woven and empowered community network can positively influence patients’ health outcomes [40]. Characterizing empowerment as the “Holy Grail of health promotion”, it is important to use this term with caution and in the right context in order to adapt to all possible situations [49].

Health is a fundamental human right that goes beyond the absence of physical and mental morbidities to a state of general well-being [58]. People are entitled to this right independent of age, gender, race, socioeconomic status, sexual orientation, and other factors [11]. There is significant and unquestionable evidence that powerlessness may result in disease [17] and that being in a position of power may lead to better health, but still, the fact that empowerment can improve health needs to be further investigated.

## 6. Women’s Empowerment and Health

As discussed earlier, powerlessness resulting from low SES is evidently a risk factor for disease, and the same can be assumed for powerlessness resulting from being a woman, within a specific societal frame. Women have been oppressed in patriarchal societies, being considered the object, with limited decision-making abilities and access to resources [3,11,26]. In a society where the reference gender is male, a significant gap in the information related to women from all aspects, including health, is frequently evident since women are not part of the equation “because when we say human, on the whole, we mean man” [59].

Therefore, it is not a surprise that the concept of empowerment was readily adopted by feminist scholars looking for tools to address and remedy the gender imbalance; hence, the term women’s empowerment appeared in the literature [32]. When the scope of empowerment is narrowed down in the context of gender, and more specifically the female gender, the importance of social structures is enhanced by the effects of years of patriarchal beliefs and the need for radical changes, either legal or political, with the support of organizations that became more eminent [4,30,32].

One of the main reasons that contribute to women suffering in silence is the fact that, due to the position of women in society, the medical knowledge available for several years in the Western world was established by male doctors with ignorance of the female body, since they had no personal experience on what women experienced physically, mentally, and emotionally. This was a legitimate bias, based on the evident differences in biology [11] that no one realized until recently, when more women were trained as physicians and researchers [12]. Consequently, the lack of information related to women was acknowledged once it became evident that men were considered the “default” gender, and women were treated as the deviation from the norm [59]. Until today, the effect of male dominance in all socio-political and economic aspects of societies has been adversely affecting women’s health, despite their recorded higher life expectancy [60]. The ways in which women’s health is being affected can be summarized as discriminatory beliefs, values, behaviors, and practices; differences in vulnerability with respect to exposure to disease; and biases in health systems and health research [11].

Being a woman is a risk factor for many morbidities, including auto-immune diseases and depression, whereas women are more prone to experiencing symptoms of pain, either in the form of headaches or migraines, endometriosis, or chronic musculoskeletal pain [12]. Also, women tend to experience more adverse drug reactions, given that for a long period, they were excluded from clinical trials of new drugs [59].

Coronary heart disease symptoms experienced by women were dismissed as psychogenic when presented in the context of stress [61], thus supporting the fact that women’s symptoms may be dismissed under the bias that “it’s all in their head”. Studies on women aged below 55 who experienced a heart attack concluded that women were reluctant to seek medical help, as they were too self-conscious that it could be a false alarm and they would be considered hypochondriacs [62]. In parallel, a meta-analysis emphasizes that when women experience heart disease, a predominantly “male” morbidity, they feel that they are not heard or seen, concluding that they are left with insufficient diagnosis and treatment [59,63].

Women often feel that they are not being heard by their physicians and that their needs are not being attended to, partly due to the social norms and biases that proclaim women to be overly dramatic in relation to their symptoms. Specifically when describing pain, women tend to be more dramatic, with the risk of their symptoms being ignored and themselves stigmatized [12]. The imbalance in social power relations between the genders results in the need to address gender in order to achieve equity in health without bias [11].

## 7. Limitations to the Study

This narrative review brings together knowledge from several fields, including feminism, gender studies, and political and social sciences, and how these interweave with social psychology, medical science, and health management, examining their intersection from a new perspective. A number of social theories mentioned in this review were solely highlighted for discussion and not explored in depth, since the scope was only to provide the historical background of oppression/repression and subsequently the emergence of empowerment theories. Political empowerment was not within the scope of this study even though the aspect of economic empowerment was discussed. Further exploration of these new connections is encouraged.

## 8. Conclusions

Women have been historically oppressed through patriarchal societies and the relevant practices that were developed from the specific beliefs in many areas including medicine. Actual or perceived oppression has been in place for so long and without anyone questioning this status since it has been considered the norm.

Empowerment, a construct that continues to challenge researchers both for its definition and measurement, may serve as a valuable tool for health promotion and women’s equity, provided it is used in the appropriate context.

Given the observed complexity and diversity in terms of how empowerment applies in different societies and cultures, one can reach the conclusion that the process of women acquiring more power is not only related to individual empowerment, which is mainly facilitated by education and economic independence, but also to their micro- and macro-social and political surroundings and the measures that can be taken toward these directions.

In order to see significant steps toward a more fair and just society, it is of major importance to keep the empowerment concept alive, in a clear and solid context toward helping people in general, and women in particular, to gain power and decision-making skills within the corresponding social and political context. Hopefully, a well-defined construct of women’s empowerment could significantly contribute to closing the gender gaps in the economy, politics, society, and health.
